# Eddy Current Testing with Giant Magnetoresistance (GMR) Sensors and a Pipe-Encircling Excitation for Evaluation of Corrosion under Insulation

**DOI:** 10.3390/s17102229

**Published:** 2017-09-28

**Authors:** Joseph Bailey, Nicholas Long, Arvid Hunze

**Affiliations:** Robinson Research Institute, Victoria University of Wellington, Lower Hutt 5010, New Zealand; Nick.Long@vuw.ac.nz

**Keywords:** eddy current testing, giant magnetoresistance (GMR) sensor, magnetic field analysis, corrosion under insulation, pipeline

## Abstract

This work investigates an eddy current-based non-destructive testing (NDT) method to characterize corrosion of pipes under thermal insulation, one of the leading failure mechanisms for insulated pipe infrastructure. Artificial defects were machined into the pipe surface to simulate the effect of corrosion wall loss. We show that by using a giant magnetoresistance (GMR) sensor array and a high current (300 A), single sinusoidal low frequency (5–200 Hz) pipe-encircling excitation scheme it is possible to quantify wall loss defects without removing the insulation or weather shield. An analysis of the magnetic field distribution and induced currents was undertaken using the finite element method (FEM) and analytical calculations. Simple algorithms to remove spurious measured field variations not associated with defects were developed and applied. The influence of an aluminium weather shield with discontinuities and dents was ascertained and found to be small for excitation frequency values below 40 Hz. The signal dependence on the defect dimensions was analysed in detail. The excitation frequency at which the maximum field amplitude change occurred increased linearly with the depth of the defect by about 3 Hz/mm defect depth. The change in magnetic field amplitude due to defects for sensors aligned in the azimuthal and radial directions were measured and found to be linearly dependent on the defect volume between 4400–30,800 mm^3^ with 1.2 × 10^−3^−1.6 × 10^−3^ µT/mm^3^. The results show that our approach is well suited for measuring wall loss defects similar to the defects from corrosion under insulation.

## 1. Introduction

Detection of the corrosion of pipe infrastructure is an important area of applied research. The annual maintenance-related expenses for the petroleum-refining industry in the USA alone is estimated to be $1.8 billion USD [1]. Usually, the equipment in processing and refinery plants is operated at elevated temperatures, so that it is necessary to insulate the pipe with a thermal insulation layer and weather shield. Corrosion under insulation (CUI) refers to corrosion on the external surface of piping and vessels underneath the insulation layer, and is one of the leading failure mechanisms for insulated pipe infrastructure. Breaks in the weather shield can lead to the ingress of water, which in combination with (cycling) processing heat leads to corrosion at the pipe surface [2]. According to an Exxon Shell study, the highest incidence of leaks in the refining and chemical industries is due to CUI and adds up to about 10% to total plant maintenance costs [3]. The consequences of not inspecting for corrosion can be catastrophic, since a pipe can rupture, which usually results in the complete shutdown of a plant or process for an extended period [4].

The simplest and most widely used inspection method is the visual inspection of the pipe surface, but this is costly as it requires the complete removal of insulation and weather shield, which also has to be reinstated after inspection. A shutdown may also be necessary if the plant cannot operate with uninsulated pipes. A tool that enables the screening of pipes without removing the insulation is, therefore, beneficial to the industry.

Radiography has been demonstrated as a fast method that allows the characterization of small defects and deposits. Its disadvantages are: only a small area can be surveyed; a complex three-dimensional geometry of source and detectors is required; access from at least one side of the pipe is needed; and strict safety requirements apply [5]. Successfully demonstrated techniques include backscatter X-ray [6], gamma ray [7], backscatter gamma ray [8,9], tangential radiography [10] and computer tomography [11].

Ultrasonic guided-wave inspection of pipes offers the possibility of rapid screening of long lengths of pipework for corrosion and other defects [12,13,14], with a typical test length being in the order of 50 m. Although only a small access area is needed, parts of the insulation have to be removed and good coupling between the transducer array and the pipe has to be guaranteed [15]. The technique needs a very good calibration procedure and is limited in its capability to determine the exact amount and location of wall loss [16]. Furthermore, it is complex to use for non-straight pipe sections [17].

A variant of ultrasonic testing uses an electromagnetic pulse instead of an acoustic pulse to generate an elastic wave that can be analysed via a second encircling coil. This technique allows evaluation without the need for a couplant. It also enables a substantial lift-off, but the technique has only been demonstrated in an experimental laboratory setup [18].

Other methods such as neutron backscatter [19] and microwave [20] work indirectly by using radiation to detect excess water and indicate potential CUI hot-spots. They are quick and accurate methods for characterizing areas susceptible to CUI and do not require the removal of insulation. Their disadvantage is that they only register smaller areas where water has accumulated under the insulation, rather than directly measuring the amount of corrosion.

Based on rerouting of directly injected currents, another recently demonstrated method injects a current into the pipe itself and measures the magnetic field change from the rerouted currents due to a defect. It has the advantage of not requiring a complex excitation scheme, but the disadvantage that the insulation has to be removed at the injection point [21].

Eddy current testing is a well-known and accepted technique for non-destructive testing and has been used to identify cracking, pitting and corrosion in metallic infrastructure [22]. By passing an alternating current through an excitation system, eddy currents are induced in the device being tested. Variations in the electrical conductivity and magnetic permeability, especially the presence of defects, cause a change in the eddy current induced and the current flow, and thus a change in phase and amplitude of the corresponding magnetic field. By using a low excitation frequency it is possible to measure through a conductive weather shield and enable the exciting field to penetrate the pipe well.

An advantage of eddy current testing is that it is a non-contact method with potentially a large stand-off distance that does not require surface preparation. A disadvantage is that changes in pipe lift-off, pipe permeability variations or discontinuities such as pipe bends or protrusions, can overshadow the defect signals or generate false positives [23].

Eddy current testing in its most basic form detects the change in magnetic field by measuring the impedance change in the excitation system itself.

Measuring the magnetic field via a magnetic sensor (array) gives spatial information that leads to better defect identification and quantification.

Giant magnetoresistance (GMR) sensors have a very low 1/*f* noise [24], which maximises the signal-to-noise ratio and enables the use of a low excitation frequency. Furthermore, due to their small size and low power consumption these solid-state thin-film magnetic sensors also allow the fabrication of compact sensor arrays enabling high spatial resolution scanning.

GMR sensors are now widely adopted for eddy current non-destructive testing (NDT) [25,26] and used for detection of small cracks [27] and subsurface cracks in aircraft structures [28], rebars in concrete [29], PCB boards [30] or support structures in steel bridges [31].

GMR sensors are also used to detect defects and corrosion in pipes. GMR sensor arrays were successfully implemented to detect cracks and defects in pipes in the pipe production process using magnetic flux leakage excitation and sensors close to the pipe surface [32,33,34]. A system based on a rotating magnetic field excitation scheme in combination with 6 GMR sensors inside a 70 mm diameter pipe with very small lift-off was able to detect defects down to 1.5 × 13.5 × 5 mm^3^ volume [35].

Another approach uses GMR sensors in combination with a meandering wire excitation scheme, targeting corrosion under insulation. It can detect corrosion patches as small as 38 × 38 × 3 mm^3^, under 50.8 mm insulation including a 0.5 mm thick aluminium weather shield [36,37].

A different form of eddy current-based technique uses pulsed eddy currents (PEC) [38,39,40]. This technique is based on the transient eddy currents following a sharp electromagnetic transition. Apart from being able to be used with a thick insulation and weather shield, the accuracy of PEC is good and it allows remaining wall thickness to be estimated. The disadvantage is that PEC averages the signal over a larger area, with dimensions typically in the range of twice the insulation thickness. Therefore, defect sizing is complex and it may miss small localised defects [38]. A pipe-encircling excitation system, similar to our approach, with a lift-off of 50 mm and 4 GMR sensors, was used to model and measure the differential PEC signal response of several defects at 4 fixed sensor positions around a pipe, the smallest defect detected having a size of 50 × 80 × 4.6 mm^3^ [41,42].

By contrast with the approaches discussed, our method uses a simple pipe-encircling, high current (300 A), single sinusoidal low frequency (5–200 Hz) excitation system in combination with a GMR sensor array. We measure the two out-of-main magnetic field components with high spatial resolution along and around the pipe to detect defect patches. In this paper we demonstrate a proof of principle measurement and analyse the change in magnetic field amplitude in an as-received pipe, a pipe with an aluminium weather shield (with and without dents in it), as well as a pipe with a set of manufactured defects. We present and discuss the correlation of the magnetic field amplitude change with excitation frequency, defect dimensions and lift-off.

## 2. Methods

### 2.1. Experimental Setup

#### 2.1.1. Pipe Mounting

**Figure 1:**
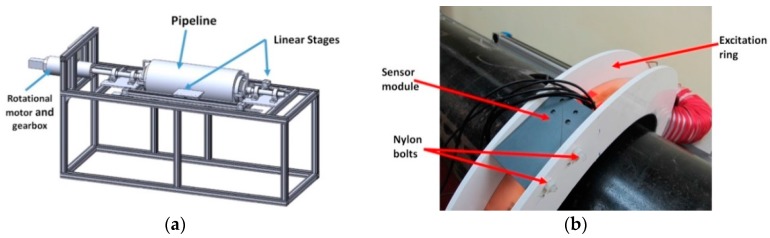
(**a**) Computer-aided design (CAD) drawing of the pipe test rig; (**b**) photograph of the excitation ring and sensor module.

A test rig was built enabling the complete scan of a pipe surface for a maximum pipe length of up to 1.3 m. The rig could support a weight of up to 100 kg. The pipe was supported at the ends allowing the excitation unit and sensor modules to move freely along the length. The CAD drawing of the system is shown in Figure 1a. The test rig included a support structure, linear motors for moving the excitation unit and sensor module along the pipe, and a rotational motor and gearbox to rotate the pipe. Due to distortions of the magnetic field at the end of the pipe, typically data was collected only within the central 700 mm. Typically, a full pipe scan of 360° around the pipe with 5° resolution and along the pipe with 5 mm resolution was undertaken; every measurement was carried out 5 times and the average values plotted as a contour map of the pipe surface.

#### 2.1.2. Magnetic Field Sensor and Excitation System

The measurement system consisted of a sensor module which is mounted directly above the excitation sheet containing the GMR sensors and off-the-shelf instrumentation for processing the data and communicating with the main LabVIEW program, run from a laptop computer.

The sensor module consisted of 3 perpendicularly aligned off-the-shelf GMR sensors from GMR sensor manufacturer NVE cooperation (Model AA004) [43]. The *z*-sensor was placed to be sensitive to field in the $\hat{z}$ direction along the pipe; the *r*-sensor was placed to be sensitive to field in the $\hat{r}$ direction perpendicular to the pipe; and the *Φ-*sensor was sensitive to field in the $\hat{\phi }$ direction around the pipe. The sensors had a linear response range between 0.5 mT and 3.5 mT and a typical sensitivity of 0.11 mV/(V·mT) [43]. Since GMR sensors were unipolar, a magnetic field biasing was necessary; this was achieved by placing small permanent magnets close to the sensors. The sensors were arranged on a concentric circle above the pipe and moved over the pipe surface.

A picture of the excitation unit coupled to a toroidal transformer is shown in Figure 1b. An encircling pipe symmetric geometry was chosen as this gives the best coupling efficiency, providing the highest ratio of input power to excitation field strength, and guaranteeing a constant magnetic field for the entire circumference. Furthermore, the setup could be moved easily along the pipe. The excitation unit was made of a stack of thin copper strips with a total thickness of 4.2 mm and was 40 mm wide. This provided a large cross-sectional area, reducing the electrical resistance and providing enough surface area for passive cooling. The overall resistance of the excitation sheet was measured as 126 µΩ. The inductance of the excitation sheet was calculated via a FEM simulation. Without a pipe, the inductance was 563 nH; with a pipe it was 763 nH (pipe material parameters, see Table 1). These values enabled a high excitation current at a low frequency. For example, for a sinusoidal peak current of 600 A at 20 Hz only a modest temperature increase of ~40 °C above ambient was measured.

#### 2.1.3. Pipe Parameters

The pipe used was a 1.3 m off-cut from a nominal 10 inch schedule 40 electrical resistance welded carbon steel pipe (carbon content below 0.3%), produced through the U-ing/O-ing process (UO pipe) [44]. It was purchased from Pipes NZ Ltd. and is typically used in New Zealand for transporting steam. The outside diameter was measured at 273 mm and the wall thickness was on average 9.1 mm measured at each end, with less than 0.3 mm variation. When mounted in the test bed, the distance from the sensor to the pipe surface varied by ±2 mm along the length of the pipe. For the pipe material, a constant relative permeability value of 63 was assumed (average value of 6 measurements described in Section 3.1). The resistivity of the pipe was measured at 170 × 10^−9^ Ω·m by cutting a small section out from the end of the pipe and performing a 4-point measurement. All pipe parameters are summarised in Table 1.

### 2.2. FEM Simulations

Apart from the experimental investigation, 3-dimensional FEM simulations using the quasi-static eddy current solver of Ansys Maxwell 3D were performed. This allowed the evaluation of a much wider parameter space and more detailed investigation of magnetic field and eddy current distribution. Since the change in magnetic field amplitude induced by a typical defect is 2–3 orders of magnitude lower than the overall strength of the magnetic field, a mesh noise-reduction technique was used: firstly, the fields were simulated with the defect set to the same material parameters as the pipe, especially refining the mesh around the defect and magnetic sensor locations. Then, a second simulation using the same mesh, but setting the defect material to vacuum, was performed. The resulting fields were then subtracted from each other. This enabled a very precise calculation of the magnetic field amplitude change due to a defect (see Section 3.2).

## 3. Results and Discussion

### 3.1. Defect-Free Pipe

The excitation used for the initial tests and simulations was set to 20 Hz, with a sinusoidal 300 A peak current and lift-off of 33 mm between excitation sheet and pipe (this lift-off distance was designed to accommodate the insulation and shield used later). Tests and simulations presented in 3.1 were done without insulation or weather shield.

#### 3.1.1. Analysis of the Magnetic Field Distribution and Induced Eddy Currents

**Figure 2:**
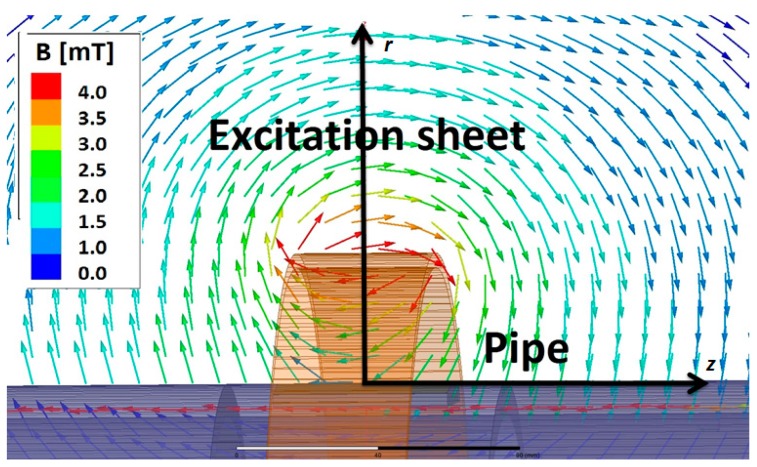
Magnetic field vector plot in *r-z* plane at 20 Hz excitation frequency.

In Figure 2, a FEM simulation of the magnetic field in the *r-z* plane in the vicinity of the excitation coil is shown. The magnetic field curls around the edges of the sheet and is forced to be parallel at the pipe surface. In the centre above the excitation sheet the field is parallel to the *z* direction. Since the GMR sensor array is located at this position, the *z*-sensor is exposed to a large background field in the *z* direction of ~4.5 mT, which leads to a saturation of the sensor. Therefore, the whole module has to be carefully positioned since any displacement from the centre of the current-carrying sheet or tilt into the *z* direction for the *r-* and *Φ*-sensors leads to increasing background field and mixing of the different field components. The magnetic field magnitude drops off quickly with increasing distance from the excitation sheet towards the pipe and has a value of ~1 mT near the pipe surface.

**Figure 3:**
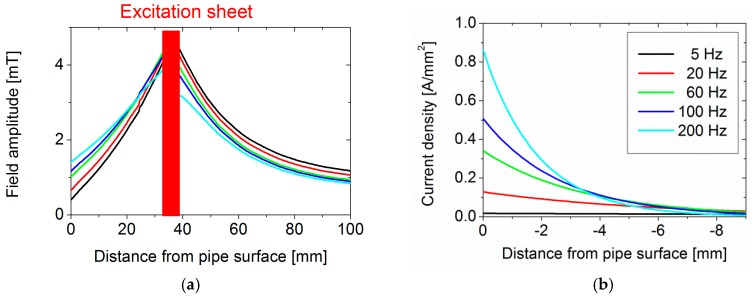
(**a**) Magnetic field magnitude in the *r-Φ* plane, through the centre of the excitation sheet versus distance from the pipe surface in the *r* direction at selected frequencies (5–200 Hz), calculated by the finite element method (FEM); (**b**) eddy current density in the pipe wall from inner radius to outer radius at selected frequencies (5–200 Hz), calculated analytically using the field values calculated by FEM.

The dependence of the magnetic field amplitude on the radial position in the *r-Φ* plane starting at the surface of the pipe is plotted in Figure 3a for different excitation frequencies. A change in excitation frequency results in a change in the magnetic field close to the sheet due to two factors: the changing current density profile in the excitation sheet, and the induced eddy currents in the pipe. The excitation sheet is made up of a stack of very thin strips of copper which means that, as the frequency increases, the current concentrates at the edge of the sheet due to lower opposition from back electromagnetic fields (EMFs) [45]. The current drops in the centre of the sheet and increases at the edge with increasing frequency. This results in a decrease in the magnetic field in the centre and an increase at the edge. A much stronger effect, however, is caused by the pipe, since induced eddy currents oppose and lower the field between the pipe and excitation sheet. Also the field lines are diverted and concentrated due to the presence of the pipe so the field amplitude decreases nearly linearly from the sheet edge to the pipe surface. Outside the pipe and sheet, the field decays as 1/*r* (see Figure 3a).

Once we know the applied field at the surface of the pipe from our FEM simulation, the induced eddy currents inside the pipe can be calculated analytically from the applied field, $\vec{H}=H_{0z}e^{i\omega t}\hat{z}$. The currents could also be calculated by FEM, but the analytical results make it easier to produce data for a wide range of geometries. By combining Faraday’s equation, Ampere’s law and the sinusoidal time dependence, it can be shown that the electric field follows Bessel’s equation,

(1) $$\frac{d^{2}E_{\phi }}{d\upsilon^{2}}+\frac{1}{\upsilon }\frac{dE_{\phi }}{d\upsilon }+\left(1-\frac{1}{\upsilon^{2}}\right)E_{\phi }=0,$$

with $\upsilon =k_{0}r\left(i+1\right)$ and $k_{0}=\sqrt{\omega \mu \sigma /2}$. The general solution for the electric field is

(2) $$E_{\phi }=A_{m}J_{1}\left(\nu \right)+B_{m}Y_{1}\left(\nu \right)$$

where *J_1_* and *Y_1_* are the Bessel and Neumann functions of first order. Likewise, the solution for the magnetic field inside the pipe is

(3) $$H_{z}=\frac{k_{0}\left(i+1\right)}{i\mu \omega }\left(A_{m}J_{0}\left(\nu \right)+B_{m}Y_{0}\left(\nu \right)\right)$$

where *J_0_* and *Y_0_* are the Bessel and Neumann functions of zero order. The constants *A_m_* and *B_m_* can be determined by applying the boundary condition that *H*_z_ must be continuous at the metal-air interfaces and using the values for the applied field amplitudes *H*_0z_ calculated by the FEM. We can then use

(4) $$J_{\phi }=\sigma E_{\phi }$$

to calculate the eddy currents.

The result for a subset of frequencies is shown in Figure 3b. This shows the expected behaviour: at higher frequencies the current magnitude decays more rapidly from the outer surface, i.e., in common terminology the skin depth is smaller. The surface current density is higher at higher frequencies in line with the higher *H*_0_ at the surface, as seen in Figure 3a.

#### 3.1.2. Measurements of Magnetic Field Distribution of a Bare Pipe

**Figure 4:**
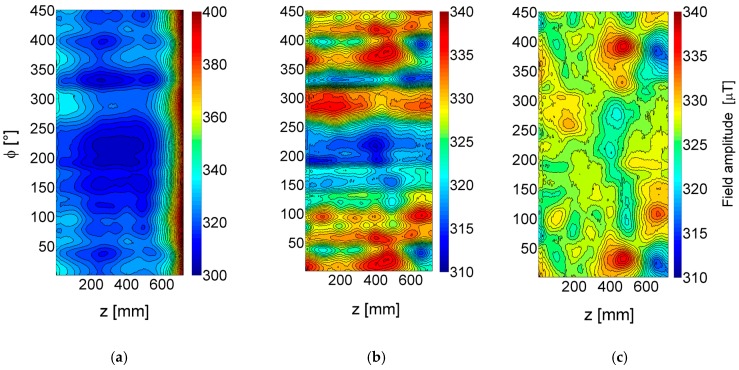
(**a**) Magnetic field amplitude over the surface of bare pipe (*r*-sensor); (**b**) same plot after applying algorithms to correct for changes in excitation current and effects at the end of the pipe; (**c**) same plot after applying an additional correction algorithm.

In Figure 4a the measurement of the magnetic field amplitude of the *r-*sensor over the pipe surface is shown, with excitation of 20 Hz and 300 A. The average value over the whole pipe surface was around 320 µT. A similar value was found for the *Φ*-sensor. The non-zero value could be attributed to a non-perfect alignment of the sensors; the value found corresponded to a small tilt (~4°) of the sensors into the main field in the *z* direction.

When the end of the pipe was reached a rapid increase in the field amplitude was observed. This was due to two different effects: firstly, as the excitation ring nears the end of the pipe, the impedance of the excitation ring drops because the amount of steel that is acting as a core decreases. This results in an increase in current and thus field, as the drive is a constant voltage. Secondly, by reaching the end of the pipe the magnetic field lines start to curl in the *r* direction increasing the field value in the *r-*sensor. This is also consistent with the finding that the *r-*sensor is more strongly affected at the end of the pipe than the *Φ*-sensor.

In order to account for this influence, a correction algorithm was developed: To account for the increasing current, the current was measured at each measurement location. This was done by stepping the driving voltage from 0.1 V to 10 V in steps of 0.1 V, fitting the current field amplitude with a linear function. This relationship was then used to correct the measured field amplitude to the nominal 300 A level for each measurement.

To account for the effect of deviation of the field at the end of the pipe, a second algorithm was applied. This algorithm first calculates the median value for each line of points around the pipe at each *z* position. The difference between this *z* position median and the median value for the whole pipe is calculated and added to all the data at this *z* position. This algorithm proved to be more effective than the current correction algorithm, suggesting that the dominant cause of the increase in field along the *z* axis is the effect of the fringe field rather than the drifting excitation current.

Once these factors are removed, the next major inhomogeneity of the magnetic field is revealed (Figure 4b): stripes of similar magnetic field amplitude along the *z* axis. The most likely source of this variation is a permeability variation around the pipe.

Overall, we expect the variations in pipe permeability to be a major influence. There is no comprehensive understanding or database available that describes how the manufacturing process and pipe usage e.g., thermal cycling, influence the permeability distribution. The literature mainly reports on permeability variations produced by a seamless production process, which induces a helical permeability variation seen in magnetic flux testing as “seamless pipe noise” [46]. Although the literature reports on failures and stress distribution of UO-produced pipes [44], we did not find reports regarding their magnetic properties.

In an attempt to quantify the variations due to permeability changes, permeability measurements were performed on 6 samples of material removed from the pipe at different *Φ* values. The value of the relative permeability varied between 20 and 128, with an average value of 63. Plotting the permeability value versus the magnetic field amplitude for the 6 different locations gave inconclusive data, possibly because drilling samples from the pipe can affect the permeability due to stress changes in the material. First, simple FEM simulations of pipes with permeability variations indicate that large area permeability variations can cause magnetic field patterns, as seen here. Furthermore, permeability gradients can produce magnetic field variations similar to defect signals, as described in 3.3.1. The same simulations as well as literature [37] indicate that, by using a multi-frequency algorithm, the response for a defect can probably be separated from the response of a permeability variation.

Nevertheless, since for this pipe the observed magnetic field variation shows a simple stripe pattern, in order to achieve a more homogeneous background a third simple algorithm was applied. Essentially, this algorithm removes the stripe pattern by normalizing the median magnetic field value for each angular position to the median overall value. This is done by calculating the median value for all points along one angular position of the pipe and then subtracting this value from the median value of the whole pipe. This correction value is then added to each data point at the angular position.

The final result of using all three algorithms can be seen in Figure 4c. Apart from a pair of positive and negative peaks centred at around 25° and 500 mm, which could be due to a local permeability gradient, a smoother background field has been achieved. The standard deviation of all data points, a good measure for remaining inhomogeneity, dropped from initially 22 µT to 2.8 µT. A similar relative improvement could be achieved for the *Φ*-sensor data. The repeatability of the measurement determined from the distribution of all amplitude values based on 5 measurements was around 10%. Therefore, in all subsequent measurements these three algorithms were applied.

### 3.2. Influence of Weather Shield

Insulated pipes in processing plants normally have a shield, providing protection from the weather as well as holding the thermal insulation in place. In most cases it is made of stainless steel or aluminium. The shielding needs to be left in place during testing, so that the weather proofing is not compromised. To evaluate the influence of a weather shield, the pipe used in the previous test was first wrapped in 25 mm mineral wool insulation. Then, a 0.5 mm aluminium sheet was wrapped around the insulation with a 300 mm overlap joint riveted along the seam with aluminium rivets. The lift-off between the excitation sheet and pipe surface was kept at 33 mm.

**Figure 5:**
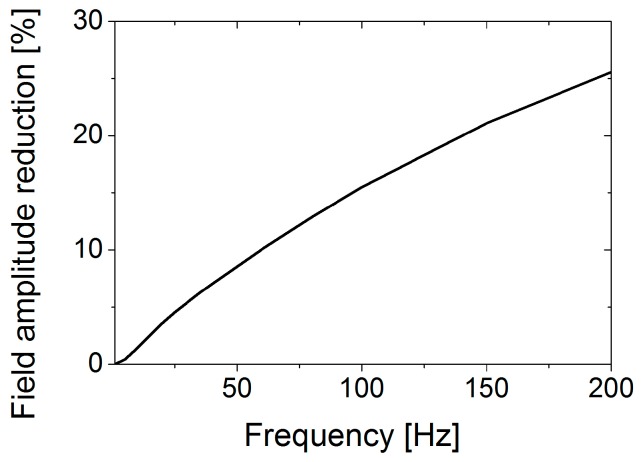
Relative reduction of the magnetic field amplitude due to a 0.5 mm aluminium shield for different excitation frequency values (FEM simulation).

In order to evaluate the reduction of the magnetic field amplitude, we simulated the effects of a 0.5 mm thick aluminium shield. Figure 5 shows the relative reduction of the magnetic field amplitude for increasing frequency values. It shows a field reduction of 25% at 200 Hz. Since this means that not only the field at the pipe is reduced considerably but also any shield inhomogeneity will create a strong eddy current rerouting and thus field change close to the sensors, we consider 200 Hz as the maximum frequency practical for our tests. Below 40 Hz the field reduction is lower than 7% and any inhomogeneity in the shield should have a smaller influence.

**Figure 6:**
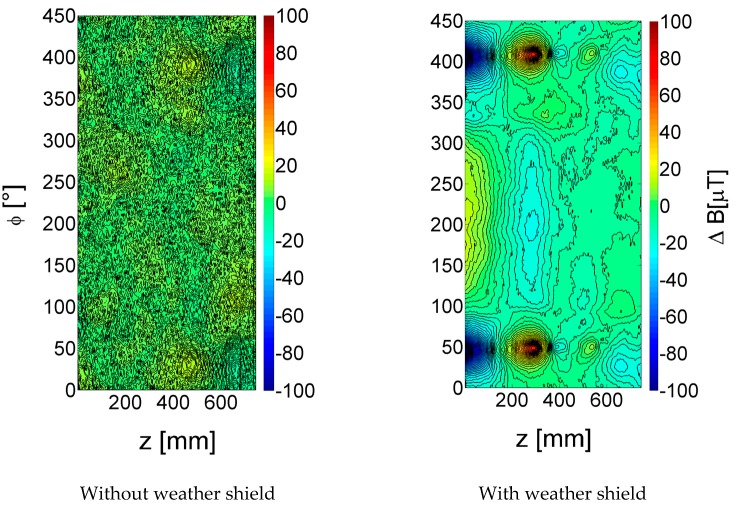
Change in magnetic field amplitude (*r*-sensor) without (left) and with (right) 0.5 mm thick aluminium weather shield; 20 Hz, 300 A excitation frequency.

In order to measure and quantify the influence of the aluminium shield, a set of tests was undertaken from 5 Hz to 200 Hz. Normalized contour plots of the *r* field amplitude data at 20 Hz with, and without the weather shield, are shown in Figure 6. Without the weather shield, a more or less smooth background was found with only a small variation around the median value as expected from the measurements discussed earlier. With the weather shield, a distinct negative and positive peak pair centred at around 50° and 200 mm with a change in field amplitude of around 200 µT peak-to-peak appeared. The signal was likely generated from the outside edge of the aluminium shield where the shield is riveted to the inside. At this point the eddy currents induced in the shield are required to make a rapid change in direction which, in turn, causes a change in the magnetic field.

**Figure 7:**
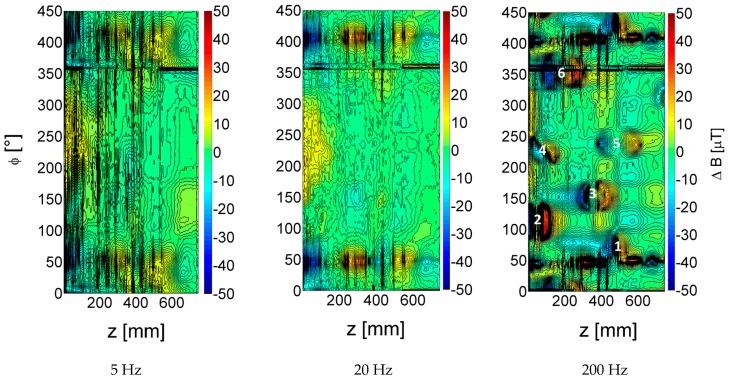
Change in magnetic field amplitude with 6 artificially introduced dents in the weather shield (*r* sensor); 5 Hz (left), 20 Hz (middle) and 200 Hz (right) excitation frequency.

In processing plants, defects and dents (e.g., due to a heavy physical impact) are often present in the shield. To investigate their influence in a second experiment, artificial dents were introduced and the same measurements performed. In Figure 7 contour plots of the *r*-sensor field amplitude change at 5 Hz, 20 Hz and 200 Hz are shown. The 5 Hz data show a noisy background and a weak indication of the shield overlap around 50°, but no clear signal from the dented aluminium, since most of the magnetic field penetrates the shield at this frequency. At 20 Hz, the signal change due to the shield overlap becomes more distinct, but still the dents cannot be observed clearly. At 200 Hz excitation frequency, a very strong signal from the shield overlap and clear features, pairs of positive and negative peaks, occur at the dents’ location (indicated by numbers 1–6). These results imply that a multi-frequency measurement is probably the most efficient way to remove the influence of the shield. This could include a high-frequency measurement, to measure the effect of any shield inhomogeneity, and then use of this data to remove the effects of the shield in the less-affected low-frequency data with a suitable algorithm.

### 3.3. Defects in the Pipe

#### 3.3.1. Measurement of Manufactured Defects

**Figure 8:**
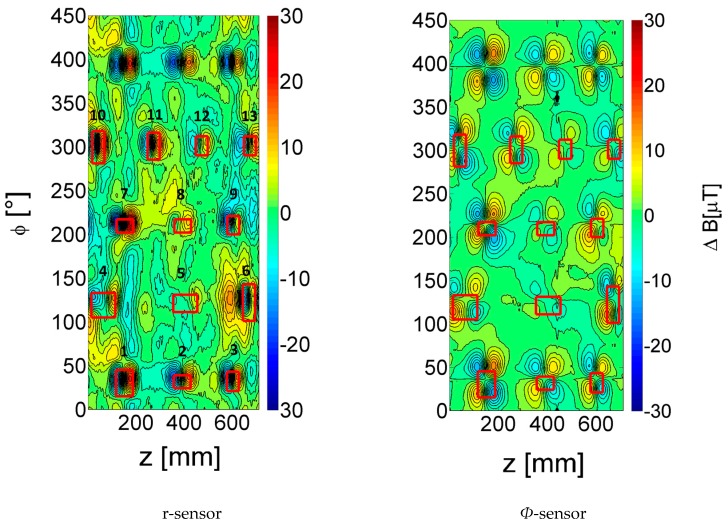
Change in magnetic field amplitude at 20 Hz and 300 A excitation current (r- and *Φ*-sensors) for pipe with defects described in Table 2.

In order to investigate signal changes due to a defect in the pipe, 13 different defects of different length, width and depth were manufactured into a similar pipe piece from the same source. The defects are rectangular and the walls were cut at constant *Φ*. The parameters of the different defects are shown in Table 2. The numbers are allocated according to their position on the pipe (see Figure 8). The position of the different defects was chosen to maximise the distance between them in order to minimise any influence from a neighbouring defect. We were informed of the sizes of the defects by our commercial partners: the defect length in *z* and *Φ* was chosen to reflect a typical extension of a corrosion patch of interest. More important though, is the capability to detect a wall loss of at least 20% of the total wall thickness; therefore, the range of defect depths was chosen between 15% and 100% of the pipe wall thickness. The following measurements as well as simulations were done without insulation and the weather shield, to exclude their influence. The lift-off of the excitation sheet from the pipe surface was kept at 33 mm.

Since the magnetic field in the *z* direction was close to 4500 µT, the *z*-sensor became saturated and it was not possible to measure a signal generated by the defect in this direction. Even if a non-saturating sensor is used, the signal change due to a defect will be very small compared to the background field e.g., the FEM simulation of defect 12 shows a change of the *B*_z_ amplitude with a maximum of around 8 µT, more than 500 times lower than the background field.

Contrary to this behaviour, *Φ-* and *r*-sensors only experience a background field due to the tilt into the main field; this means the expected background field is much lower and easier to account for, as discussed in Section 3.1.

The two-dimensional plot of the change in magnetic field amplitude of the *r-* and *Φ-*sensors at an excitation frequency of 20 Hz and current of 300 A is shown in Figure 8. The 20 Hz data showed the least noisy defect patterns and, as described in Section 3.3.4, this frequency value is closest to the frequency value of the best signals expected for most of the defects in Table 2. The defect outer edges are indicated by red boxes. All defects show a clear signal especially for the *Φ-*sensor. Only defect 5, an elongated defect in the *z* direction and having the lowest defect depth, is harder to identify visually in both sensor plots.

All defects including the through holes (defects 3 and 7) show a similar pattern. The *r-*sensor signal exhibits a dipole profile, while the *Φ*-sensor has a quadrupole-like pattern.

The specific shape can be explained by the path of rerouted currents around the defect. In a defect-free infinite pipe, the induced current flows solely around the pipe in the *Φ* direction. By introducing a defect, a deflection of the current will occur in the *z* and *r* directions, prevalent near the edges of the defect. We will concentrate our discussion on the current deflected in the *z* direction as our simulations show the effect of additional current in the *r* direction on the out-of-main field sensors is small.

According to Bio Savart’s law, the magnetic field $d\vec{B}$ generated by a current carrying element $id\vec{l}$ can be described as:

(5) $$\begin{array}{ccc} d\vec{B} & = & \frac{\;id\vec{l}\times {\vec{e}}_{R}}{4\pi R^{2}} \end{array}$$

**Figure 9:**
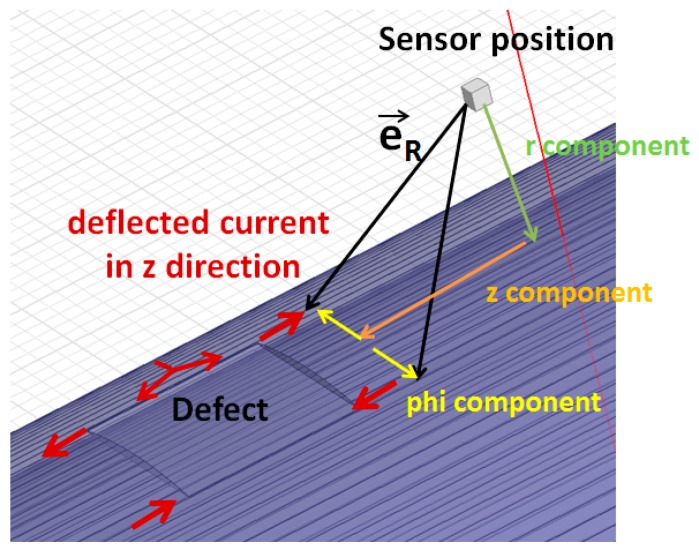
Simplified schematic of rerouted eddy currents in surface of pipe (z components) and directional vector deconvolution.

*i* is the current amplitude in distance element dl, *R* is the distance from the current element to the sensing point, and ${\vec{e}}_{R}\;$is the directional unit vector along the line between the element and sensing point (see Figure 9).

The simplest way to understand the resulting pattern is using the right-hand rule expressed in Bio Savart’s equation: the redirected current in the ±*z* direction will introduce a magnetic field in the *r* and *Φ* directions as follows: $d{\vec{B}}_{r}\propto i\hat{z}\times \phi \hat{\phi }$ and $d{\vec{B}}_{\phi }\propto i\hat{z}\times r\hat{r}$.

The current elements closest to the sensor have the largest effects. For the *r*-sensor, the components have two possible causes of reversed polarity. The first is the reversed current at the edges of the defect due to the described current flow. The second is the reversed direction of the *Φ* component as the sensor position is at positive or negative *Φ* relative to the current element. For a constant *Φ*, the sensor response changes polarity as the sensor moves along the z direction over the defect due to the changing current direction. When the sensor moves in the *Φ* direction over the defect, *Φ* reverses and the current in the *z* direction also reverses so there is no change in polarity of *dB*_r_. The net effect is a dipole pattern, with poles separated in the *z* direction.

The magnetic field in the *Φ* direction is proportional to the current element in the *z* direction and the *r* component of the directional vector. The only change of polarity is due to the reversed currents, and the outcome is a quadrupole pattern.

Altogether, this explains the observed dipole pattern for the *r* and quadrupole pattern for the *Φ*-sensor.

#### 3.3.2. Defect Signal Strength Experimental Data

**Figure 10:**
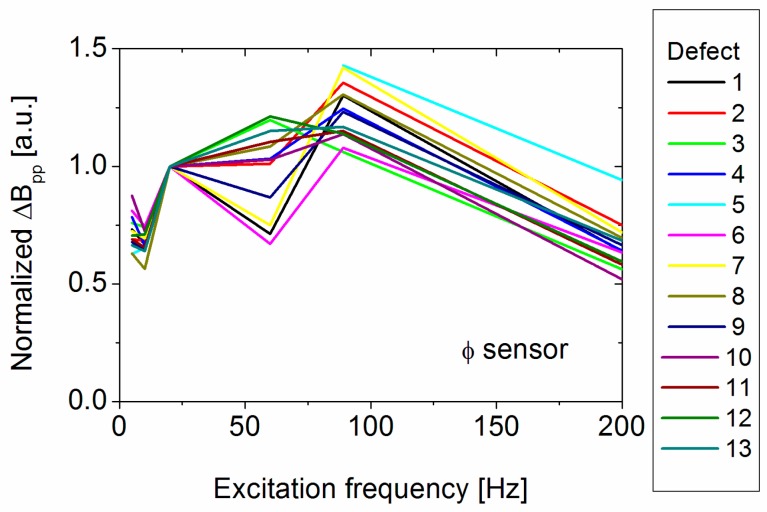
Measured excitation frequency-dependence of the 20 Hz normalized peak-to-peak change in magnetic field amplitude for the *Φ*-sensor.

To investigate the defect signal in more detail, a set of measurements at 5, 10, 20, 60, 89 and 200 Hz were performed. The excitation current was, as before, 300 A. Figure 10 shows the frequency dependence of the peak-to-peak change in magnetic field amplitude, Δ*B*_pp_ for the *Φ*-sensor for all defects normalized to the 20 Hz value. The *r*-sensor shows a similar behaviour but has a larger data spread. The *Φ*-sensor data also show a quite large data spread. Integrating the total defect signal instead of using only the peak-to-peak value leads to a similar curve. Apart from an increase of the signal above 10 Hz and a decrease for 200 Hz, and a maximum in between these values, no general trend can be observed. This is probably due to considerable measurement noise, possibly induced by pipe material variations and sensor alignment.

**Figure 11:**
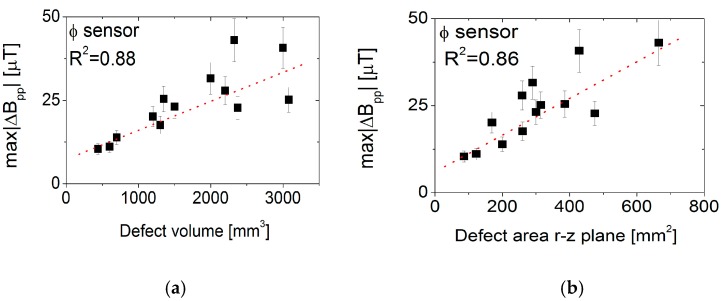

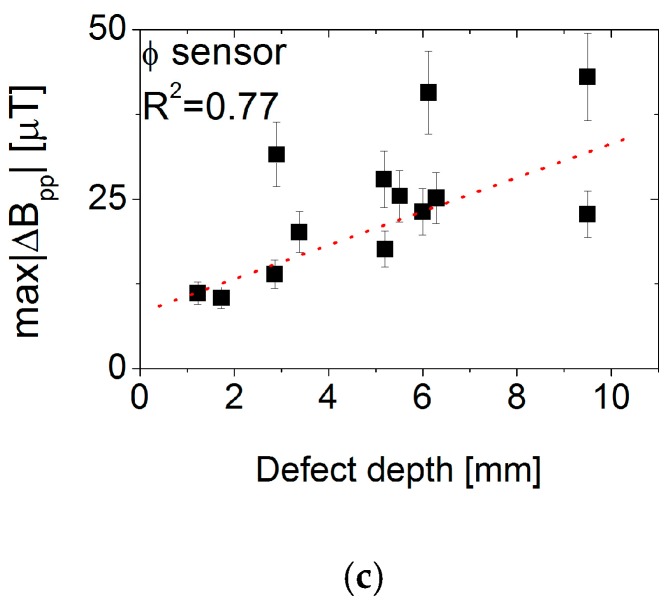
Dependence of maximum peak-to-peak change in magnetic field amplitude on (**a**) defect volume, (**b**) defect area in the *r*–*z* plane, and (**c**) defect depth.

Further analysis of the defect signal strength is shown in Figure 11, where major defect parameters—defect volume, defect depth and cross sectional area in the *r-z* plane—are plotted versus the maximum peak-to-peak change in magnetic field amplitude, $\max\left|\Delta B_{pp}\right|$, over all frequencies.

All data show a more or less linear dependence of $\max\left|\Delta B_{pp}\right|$ on the dimensional parameter, as indicated by the dotted line. Due to the large data spread, the R^2^ values are quite low and similar; and, since the manufactured defects have increasing volume with increasing cross-sectional area and increasing defect depth, from the measurement data it cannot be determined if one of the defect parameters has a dominant influence.

#### 3.3.3. Validation of FEM Simulations

**Figure 12:**
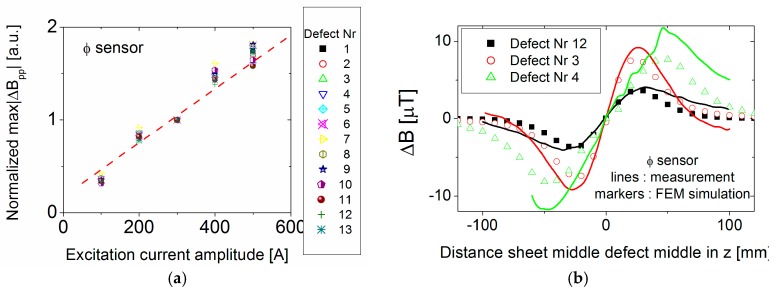
(**a**) Maximum peak-to-peak change in magnetic field amplitude for the *Φ*-sensor normalized to 300 A; (**b**) comparison of measured and FEM simulation results of change in magnetic field amplitude for the *Φ*-sensor located at the defect edge and moving over the defect in the *z* direction.

To investigate the dependence of the defect signal in more detail, FEM simulations were performed. Since the excitation current of 300 A was quite large, and the FEM solver assumes a linear constitutional behaviour between magnetic field and excitation current, an experimental test was performed first. In Figure 12a, the measured relationship between $\max\left|\Delta B_{pp}\right|$ normalized to the 300 A value for the *Φ*-sensor is shown for all defects for an excitation current between 100 A and 500 A. This shows a linear relationship with a relative increase of ~20% every 100 A. This means that the assumption of linear constitutional behaviour of magnetic field amplitude and excitation current in the FEM solver is valid.

In order to validate the FEM simulations, a direct comparison of the simulated and measured defect signals was made. In Figure 12b, the *Φ*-sensor signal at 20 Hz excitation frequency and 300 A excitation current along the *z* axis is shown for 3 different defects: the defect with the lowest volume (Nr 12), the corresponding through hole (Nr 3), and a large defect with average depth extended in the *z* as well as the *Φ* directions (Nr 4). It can be seen that there is reasonable agreement between FEM simulations and experimental data. The average deviation between measured and simulated data is between 20% and 40%, and other simulated defects and the *r-*sensor data show a similar agreement. The deviations are likely due to local variations of permeability around the defect; the FEM simulations assumed a constant permeability of 63. Nevertheless, these data show that the FEM simulation can be used to determine the influence of defect parameters.

#### 3.3.4. Frequency-Dependence of Defect Signal

**Figure 13:**
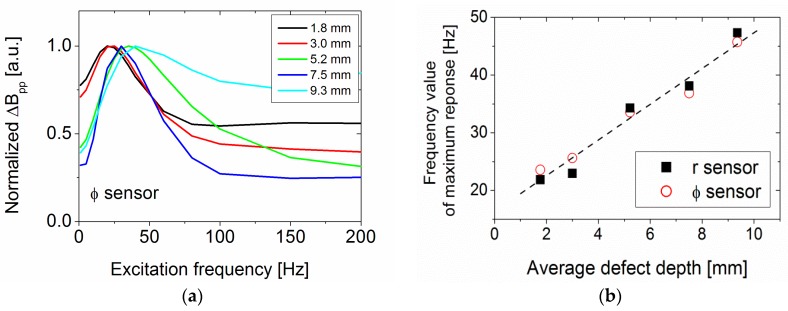
FEM simulations (**a**) *Φ*-sensor: normalized maximum peak-to-peak change in magnetic field amplitude versus frequency for defects with increasing average defect depth; (**b**) frequency of maximum response from (**a**) versus defect depth.

To investigate the dependence of the field amplitude change with excitation frequency, defects of 50 mm length in the *z* direction, 21° in the *Φ* direction, and different defect depths were simulated for excitation frequencies between 1 and 200 Hz. The normalized maximum peak-to-peak change in magnetic field amplitude for the *Φ*-sensor is shown in Figure 13a. The *r*-sensor behaves similarly.

As the measured curve shows, the signal increases until a maximum response between 20 Hz and 40 Hz is reached and then decreases or levels off at higher frequency values. But in contrast to the measured curve, the simulated data show a clear maximum, which depends on the average defect depth (see Figure 13b). The frequency value of maximum sensor response increases linearly with increasing average defect depth, with ~3 Hz per mm average defect depth for both sensors.

**Figure 14:**
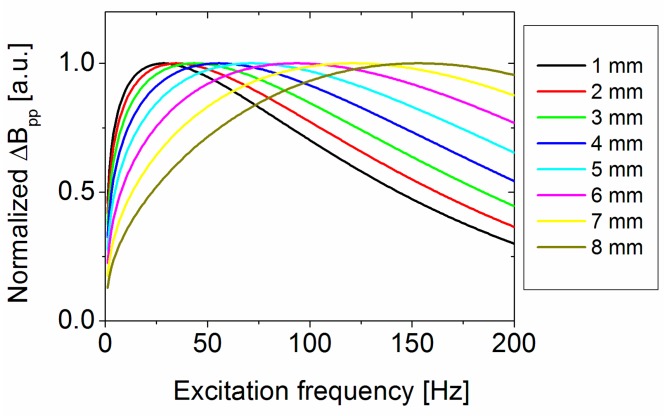
Normalized peak-to-peak change in magnetic field amplitude versus frequency for defects with increasing defect depth using Formula (6).

This behaviour can be explained qualitatively with a simple model: as discussed in 3.1, the change in field amplitude is due to the rerouted eddy currents. We constructed a simple model which assumes the rerouted eddy currents are proportional to the current lost due to the presence of the defect. The eddy current lost due to the defect is proportional to the difference of the eddy currents without a defect and the eddy currents induced in the remaining wall thickness. Since the extension in the *Φ* and *z* directions in the calculation is the same, only the difference in the *r* direction needs to be accounted for. Neglecting the larger increased lift-off from the defect surface, this can be calculated to:

(6) $$\int_{0}^{p}j\left(s,f\right)\;ds\;-\;\int_{0}^{p-d}j\left(s,f\right)\;ds=\int_{p-d}^{p}j\left(s,f\right)\;ds$$

where j(s,f) is the frequency, *f*, dependent current density inside the pipe wall calculated by (4), *s* is the distance from the outer pipe wall, *p* is the pipe wall thickness, and *d* is the defect depth. A plot of the resulting curve for different defect depth values is shown in Figure 14.

The calculated curve shows a qualitatively similar dependence as the FEM simulation in Figure 13a: increasing field amplitude until a maximum is reached, and levelling off at higher frequency values. Also, the maximum of the response shifts with increasing defect depth. The absolute value of the frequency where the maximum occurs as well as the increase per mm defect depth is higher than the FEM simulated response, especially for deep defects; the curve is also wider. There are probably several reasons for the deviation: the surface current at the bottom of the defect is not only dependent on the increased distance from the excitation sheet but also the surrounding remaining material. Notably, the proportion of the induced current that is redirected around the defect probably depends on the exact shape and remaining wall thickness in a more complex way, which means this simple model can only explain qualitative behaviour.

#### 3.3.5. Influence of Defect Dimension on Signal Strength

Since from the experimental data the influence of the defect dimensions in the *r*, *Φ* and *z* directions could not be determined, another set of FEM simulations was performed.

**Figure 15:**
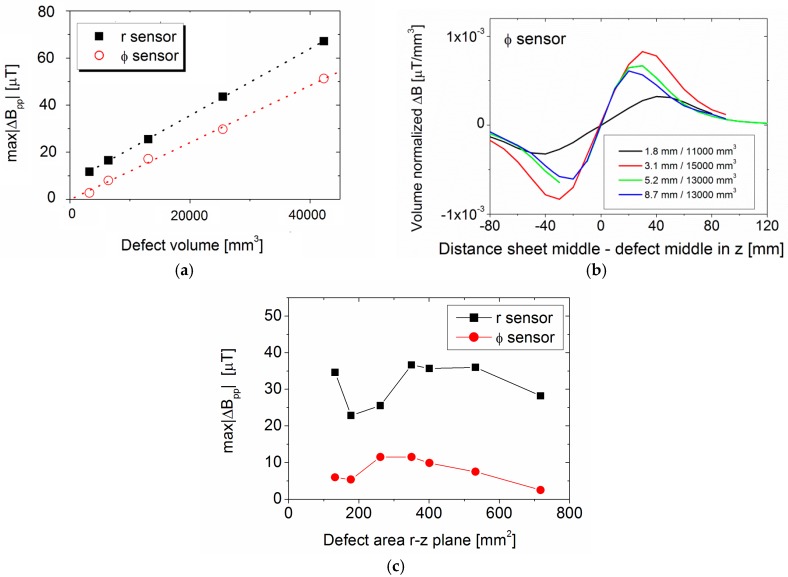
FEM simulations (**a**) maximum peak-to-peak change in magnetic field amplitude for defects with constant average depth but varying defect volume; (**b**) defect volume normalized change in magnetic field amplitude of *Φ*-sensor signal for defects with similar volume but different average defect depth; (**c**) maximum peak-to-peak change in magnetic field amplitude for defects with constant volume and constant average depth but changed cross-sectional area.

In order to distinguish the influence between defect volume and defect depth, a FEM simulation was undertaken of 5 rectangular defects with a constant average depth of 5.2 mm but different defect size in the *Φ* and *z* directions and thus different volumes, in a similar range as the measured defects. The frequency of the maximum signal occurred at the same frequency value at around 30 Hz, as expected from simulations discussed before. A linear dependence between the defect volume and *Φ* and *r* peak-to-peak field amplitude change was found, with 1.2 × 10^−3^ µT/mm^3^ for the *Φ*-sensor and 1.4 × 10^−3^ µT/mm^3^ for the *r*-sensor (see Figure 15a). This agrees well with the experimental data: 1.3 × 10^−3^ µT/mm^3^ for the *Φ*-sensor and 1.6 × 10^−3^ µT/mm^3^ for the *r*-sensor. The simulated defect volumes change by 1320%, the defect signal change spans a similar range: *r* signal 570% and *Φ* signal 2000%.

A second simulation was undertaken, keeping the volume in a narrow range of 11,000–15,000 mm^3^ but varying the defect depth between 1.8 mm and 8.7 mm. The volume normalized magnetic field amplitude change of the *Φ*-sensor at the defect edge was very similar for all depths, apart from the very large and shallow defect, as shown in Figure 15b. This means that the defect depth alone only has a small influence on the signal strength.

In order to distinguish between the influence of the defect volume and the defect cross-sectional area, a third simulation was undertaken. The volume was kept constant to 13,200 mm^3^ and the average defect depth constant to 5.2 mm. The cross-sectional area was changed within the same range as the measured defects between 100 mm^2^ and 700 mm^2^. In Figure 15c, the cross-sectional area in the *r*–*z* plane is plotted versus the maximum peak-to-peak magnetic field amplitude change. A more complex non-linear influence exists, but the defect signal variation is only 60% for the *r*-sensor and 115% for the *Φ*-sensor, while the cross-sectional area changes more than 700%. This more complex behaviour is probably partially due to the finite sheet width, which is exciting eddy currents over different fractions of the defect length in the *z* direction.

Taken together, all results indicate strongly that the signal strength is not determined by only defect depth or a cross-sectional area but rather by the overall defect volume. This is also in agreement with the simple model explaining the frequency behaviour, which was discussed earlier.

#### 3.3.6. Influence of Lift-Off Distance

**Figure 16:**
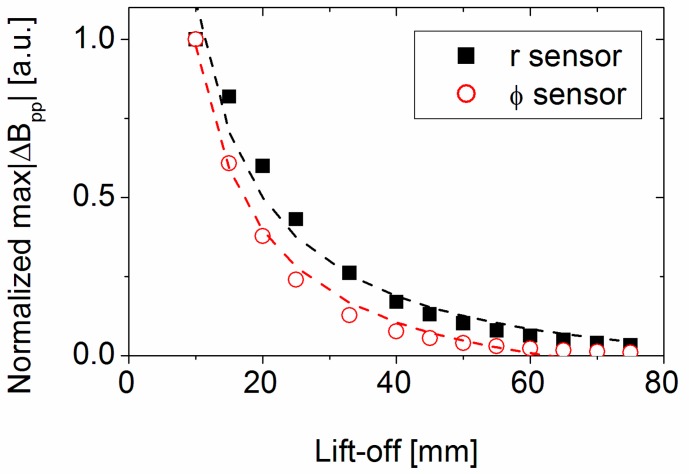
Normalized lift-off dependence of maximum peak-to-peak change in magnetic field amplitude for *r-* and *Φ*-sensors for defect Nr 12 (FEM simulation).

The last important parameter regarding signal strength is the dependence of the defect signal on the lift-off between the excitation sheet and sensor unit and pipe. The normalized (to 10 mm lift-off) maximum peak-to-peak change in magnetic field amplitude for defect Nr 12, with increasing lift-off between pipe surface and excitation/sensor unit, is shown in Figure 16. Both sensors show a strong decrease with increasing lift-off, which reflects the decrease in exciting field at the pipe surface. The curves can be described by a 1/*r* dependence (shown as dotted lines).

## 4. Conclusions

We presented an eddy current-based testing scheme to evaluate corrosion under insulation in pipes. It is based on a GMR sensor array and a pipe-encircling high current, single sinusoidal excitation system. The excitation setup provides good coupling with the pipe and symmetric excitation around the pipe. Due to low 1/*f* noise of the GMR sensors a low excitation frequency (down to 5 Hz) can be used, which enables magnetic field penetration through the aluminium weather shield and strong field penetration into the pipe itself.

A defect-free, UO-processed nominal 10 inch schedule 40 electrical resistance welded pipe shows stripes of constant magnetic field amplitude around the pipe that can be compensated for by a simple algorithm.

The influence of an aluminium weather shield was investigated at different excitation frequency values, with and without dents in the shield. For higher excitation frequencies, a clear signal from the dents could be observed, which diminishes with decreasing frequency and correlates with the field reduction due to eddy currents induced in the shield. By using a low frequency excitation, it is possible to penetrate the aluminium shield with only minimum signal from any distortions in the shield itself. Interfering signals from the shield can probably be further reduced by using a multi-frequency algorithm to measure and then subtract the interfering signal.

Due to the high field near the excitation sheet, the GMR sensor aligned in the main field direction becomes saturated very quickly. Also, the expected signal in the main *z* direction due to a typical defect is small compared to the background field.

Sensors in the axial *r* and azimuthal *Φ* direction show a lower background field, caused only by the misalignment of the sensor from the centre position. These sensors show a clearly visible change of magnetic field amplitude due to artificially manufactured defects to simulate corrosion wall loss in the pipe. The defect pattern in the *r* direction has a dipole profile, the pattern in the *Φ* direction a quadrupole profile. This pattern can be explained by the rerouting of the induced current around the edges of the defect, especially the eddy current component in the *z* direction.

The frequency dependence of the magnetic field amplitude change shows a maximum response between 20 and 50 Hz and is increasingly linearly dependent upon the defect depth. Measurements as well as simulations show that the peak-to-peak value is mainly dependent on the defect volume with a linearly increasing response of 1.2–1.6 × 10^−3^ µT/mm^3^.

Both observations can be qualitatively explained by the assumption that field change is proportional to the eddy current rerouted in the presence of the defect, and the rerouted current is proportional to the eddy current lost due to the defect.

Increasing insulation thickness means increasing lift-off between the pipe surface and excitation sheet leads to a decreased defect signal and can be described with a 1/*r* dependence.

Taken together, the results suggest that our approach is well suited for measuring and quantifying corrosion under insulation. Based upon our concept, a field-ready prototype tool including a multi-frequency measurement, targeting insulation thickness of up to two inches, is being built and tested.

## Figures and Tables

**Table 1:** Pipe parameters.

Parameter	Value
Length	1.3 m
Inner diameter	254.8 mm
Outer diameter	273 mm
Resistivity	170 × 10^−9^ Ω·m
Relative permeability	63

**Table 2:** Parameters for defects manufactured into surface of pipe.

Defect	Defect Volume (mm^3^)	Defect Length in *z* (mm)	Defect Length in *Φ* (°)	Average Defect Depth (mm)
1	30,000	70	29	6.1
2	13,500	70	14.5	5.5
3	23,800	50	21	9.3
4	20,000	100	29	2.9
5	6000	100	20	1.2
6	30,800	50	41	6.3
7	23,300	70	16	9.3
8	7000	70	16	2.9
9	15,000	50	21	6.0
10	22,000	50	35	5.2
11	12,000	50	30	3.4
12	4400	50	21	1.7
13	13,000	50	21	5.2
